# Risperidone or Aripiprazole Can Resolve Autism Core Signs and Symptoms in Young Children: Case Study

**DOI:** 10.3390/children8050318

**Published:** 2021-04-22

**Authors:** Hamza A. Alsayouf, Haitham Talo, Marisa L. Biddappa, Emily De Los Reyes

**Affiliations:** 1 Kids Neuro Clinic and Rehab Center, Dubai Healthcare City, Al Razi Medical Complex, Dubai 1015, United Arab Emirates; htalo@yahoo.com (H.T.); marisa.lobobiddappa@gmail.com (M.L.B.); 2 Pediatric Neurology, Nationwide Children’s Hospital, Ohio State University, Columbus, OH 43210, USA; Emily.delosReyes@nationwidechildrens.org

**Keywords:** autism spectrum disorder, risperidone, aripiprazole, antipsychotic agents, case study

## Abstract

Risperidone and aripiprazole are approved by the USA Food and Drug Administration for the treatment of irritability and aggression in children from the ages of 5 and 6 years, respectively. However, there are no approved medications for the treatment of autism spectrum disorder (ASD) core signs and symptoms. Nevertheless, early intervention is recognized as key to improving long-term outcomes. This retrospective case study included 10 children (mean age, 2 years 10 months) with ASD who presented with persistent irritability and aggression before 4 years of age that was unresponsive to behavioral interventions and sufficiently severe to consider pharmacological intervention with risperidone or aripiprazole combined with standard supportive therapies. Besides ameliorating comorbid behaviors, improvement was observed in ASD core signs and symptoms for all patients, with minimal-to-no symptoms observed in 60% of patients according to the Childhood Autism Rating Scale 2-Standard Test and Clinical Global Impression scales. Excessive weight gain in two patients was the only adverse effect observed that required intervention. This is the first study to suggest that ASD can potentially be treated in very young children (<4 years). Clinical trials are urgently required to validate these findings among this pediatric population.

## 1. Introduction

Autism spectrum disorder (ASD) is a complex and highly heterogeneous neurodevelopmental disorder, with each patient being unique in their presentation [1]. However, there are core symptoms that are universal to all patients, including impaired communication and social interaction, and restricted and repetitive behaviors. ASD is increasing in prevalence, with 1 in 54 children diagnosed in the USA in 2016 compared with 1 in 150 in 2000 [2]. This increase, at least in part, may be due to improved detection methods and increased awareness among parents and clinicians [3,4].

ASD is an umbrella term that has replaced older terminology for autistic disorder, Asperger’s syndrome, childhood disintegrative disorder, and pervasive developmental disorder not otherwise specified [1,5]. ASD symptoms can appear before 2 years of age but typically become more evident between 2 and 3 years of age [1,6]. Children with ASD experience developmental difficulties with behavior, communication, and socialization to a variable and often debilitating extent. Although core domains can improve with age, several symptom subdomains, including limited interests, social smiling, and emotional expression, may remain unchanged with poor outcomes common in later adulthood [7,8,9,10].

The standard recommended treatment of ASD in children involves early intervention with behavioral, occupational, and speech therapies to support healthy development and improve socialization [5]. No medications have yet demonstrated efficacy in clinical trials and been approved by any regulatory agency for the treatment of ASD core signs and symptoms [11]. Instead, medications are prescribed where needed as an adjunct to treat comorbid challenging behaviors so as to support children with their development and social functioning [12,13,14].

Risperidone and aripiprazole were approved by the USA Food and Drug Administration (FDA) in 2006 and 2009 [15,16] for the treatment of irritability and aggression in children from the age of 5 or 6 years for risperidone or aripiprazole, respectively [17,18], and these remain the only medications approved in the USA for treating any ASD-associated comorbid behavioral problems [19]. Other common challenging comorbid behaviors in children with ASD include self-injury, hyperactivity, and disruptive behavior. Improvements in comorbid behaviors are frequently observed in children treated with risperidone or aripiprazole [20]. However, these drugs are mixed dopamine- and serotonin-receptor antagonists or partial agonists, falling within a class of medications termed atypical antipsychotics [14]. Atypical psychotics can cause adverse effects such as weight gain, hyperprolactinemia, sedation, cardiac symptoms (e.g., tachycardia or a prolonged QTc interval), extrapyramidal symptoms (e.g., dystonia or tardive dyskinesia), and metabolic disturbances (e.g., hyperlipidemia or hyperglycemia) [11,21,22]. Therefore, children treated with these medications require close monitoring with regular follow-up.

Several medications have been prescribed to mitigate medication side effects or help improve symptoms in older children with ASD when medication switch or discontinuation is not an option. For example, metformin may be prescribed to manage weight gain induced by risperidone [23], whereas methylphenidate, atomoxetine, guanfacine, and bupropion, which are used to treat attention-deficit hyperactivity disorder (ADHD), have been used to alleviate hyperactivity and poor attention in older children with ASD [24,25,26,27]. However, the addition of further medications needs to be carefully weighed as these increase the burden of potential adverse effects; ideally, medications causing adverse effects should be discontinued.

ASD is currently considered a non-curable disorder [1], and no registered clinical trial of risperidone or aripiprazole has provided evidence for their use in treating the core ASD signs and symptoms in children, even though there have been many studies on their use in children aged 5–18 years for the treatment of comorbid challenging behaviors [11,20]. For risperidone, there have also been several reports of the treatment of children with ASD under the age of 4 years [28,29,30,31,32,33,34,35,36,37]. Importantly, some of these studies have noted improvements in not only comorbid challenging behaviors but also social relatedness, restricted and repetitive behaviors, and levels of cooperation with developmental activities.

In this report, we therefore present a single-center, retrospective case study describing our experience in treating 10 very young children with ASD and challenging comorbid behaviors. These children initiated treatment between the age of 2 to 4 years with risperidone or aripiprazole to manage persistent comorbid challenging behaviors, either with or without atomoxetine or methylphenidate, and in combination with standard supportive therapies.

## 2. Patients and Methods

### 2.1. Patients and Data Collection

This was a retrospective case study of all patients diagnosed with ASD as per the Diagnostic and Statistical Manual of Mental Disorders, 5th edition (DSM-5) criteria and the Childhood Autism Rating Scale 2-Standard Test (CARS2-ST), who started treatment at our center between the age of 2 and 4 years for persistent comorbid behavioral issues (Kids Neuro Clinic and Rehab Center, Dubai, United Arab Emirates) from 2016 to the end of 2019, who had not responded to behavioral interventions, and who met the following inclusion criteria: (1) normal electroencephalogram with sleep sample, (2) normal auditory brainstem response (ABR) hearing test, (3) no other associated chronic medical condition, (4) no underlying genetic disorder, (5) compliance with our treatment protocol, (6) no associated global developmental delay, (7) Clinical Global Impression-Severity (CGI-S) score ≥ 4 at the time of presentation, (8) had not been previously treated with any antipsychotic medication, and (9) the family agreed to participate in our study and the patient’s parents/guardian provided written informed consent. There were 19 children with ASD treated with aripiprazole or risperidone at our center during this period who were less than 4 years and older than 2 years old, but only 10 children met all the above-mentioned criteria. Five patients were noncompliant with the treatment protocol, two patients had underlying genetic disorders, and two patients had associated global developmental delay. This study was approved by the Dubai Healthcare City Regulatory Ethics Committee: Approval no. KNCRC-02.

The following data were collected for all patients: age at presentation, age at treatment initiation, medications used, starting dose, time to reach the maximum dose, current medications and doses, adverse effects, analyses performed, initial signs and symptoms, initial formal diagnosis as per the DSM-5 criteria and CARS2-ST, baseline CARS2-ST and CGI-S scores before starting medications, CGI-I scores after treatment, and final CARS2-ST and CGI-S scores as per their last visit to our clinic. Specific data on the socioeconomic status and educational attainment levels of the patients’ families were not recorded.

The CGI-S and CARS2-ST were used as a baseline for rating each patient’s severity prior to initiating medication. Severity was scored with the seven-point CGI-S scale: 1 = normal, not at all ill; 2 = borderline, mentally ill; 3 = mildly ill; 4 = moderately ill; 5 = markedly ill; 6 = severely ill; 7 = among the most extremely ill patients [38]. CARS2-ST can be applied to children of 2 years and older and uses a 15-category scoring system to classify patients into one of three ASD severity groups: minimal-to-no symptoms of ASD, mild-to-moderate symptoms of ASD, and severe symptoms of ASD [13,39,40].

CGI-I was used to measure the overall improvement of each patient after treatment initiation. CGI-I is scored based on a seven-point scale that compares the patient’s current and baseline condition (initiation of treatment): 1 = very much improved since the initiation of treatment; 2 = much improved; 3 = minimally improved; 4 = no change from baseline; 5 = minimally worse; 6 = much worse; 7 = very much worse since the initiation of treatment [38].

### 2.2. Diagnosis

A thorough history and physical examination were performed for each patient presenting at our clinic with signs and symptoms suggestive of ASD. An electroencephalogram (EEG) with a sleep sample was obtained to identify electrical status epilepticus in sleep or epileptic encephalopathy, and an ABR hearing test was conducted to detect any hearing loss. These tests aimed to exclude conditions necessitating other treatment approaches [41,42,43,44]. Pharmacological treatment was recommended if there was a normal ABR, normal EEG, no findings suggesting a genetic disorder (e.g., family history of genetic disorders, dysmorphic features, or an abnormal genetic test result), no global developmental delay, an ASD diagnosis based on the DSM-5 criteria and CARS2-ST, and a CGI-S score ≥ 4 with persistent comorbid behavioral issues. Diagnoses were made by a pediatric neurologist and a clinical psychologist following multidisciplinary assessment.

### 2.3. Treatment

Overall, we followed the same treatment protocol used for children older than 4 years published in our previous study [45]. For persistent comorbid behavioral symptoms, patients were prescribed either risperidone or aripiprazole [15,16]. Because risperidone has been more extensively studied among children with ASD, it was suggested as the first-line treatment; however, some families opted for aripiprazole instead. The children’s parents and legal guardians were counseled regarding the off-label use and potential side effects of the antipsychotic medications. As this study was a retrospective case study, no control (placebo) treatment or randomization were performed. Risperidone was typically initiated with 0.25 mg orally at night and gradually increased every 1 to 2 weeks to the maximum dose (2 mg, morning; 0.5 mg, nighttime) or the maximum dose tolerated. This approach was initiated for 8/10 cases (all except cases 2 and 4). For families that chose to initiate aripiprazole treatment (cases 2 and 4), patients were started on 1 mg at night and this dose was gradually increased every 1 to 2 weeks to the maximum dose (10 mg, nighttime) or the maximum dose tolerated. The regimens for risperidone and aripiprazole were adhered to while the patient showed improvement with each dose increment and had no adverse effects. Table 1 outlines the patients’ dosing schedules and relevant clinical data.

When a patient tolerated their medication without adverse effects and with progressive improvement to a CGI-I score of 1 or 2, the medication was continued. If their CGI-I plateaued at a score of 2 or higher, or there was excessive weight gain and persistent hyperactivity or inadequate attention span, methylphenidate or atomoxetine was added to augment improvement to a CGI-I score of 1, improve attention, and control excessive weight gain. Further, all children with progressive weight gain were referred to a nutritionist to minimize the development of other health issues, non-compliance, and premature weaning off of their medication.

Methylphenidate was usually added at 2.5 mg in the morning and increased gradually to a maximum total daily dose of 20 mg or less as tolerated (this maximum dose was only reached for case 5 who was almost 4 years old). Atomoxetine was typically started at 10 mg at night and increased gradually to a maximum dose of 2 mg per kg mg or less as tolerated if no adverse effects were observed and an improvement was seen with each dose increment (Table 1). When a patient reached a CGI-I score of 1 and a repeat CARS2-ST score of minimal-to-no symptoms, the medications were continued for a further 6 months before attempting to slowly wean off all medications.

All patients were strongly encouraged to continue with standard supportive therapies such as speech therapy, applied behavior analysis therapy, and occupational therapy. Since most patients were from families with limited resources, all received on average four to six sessions a week of speech and applied behavior analysis therapies, except for case 7 whose family was unable to afford any therapies or even daycare. Parents and caregivers were given practical support and guidance and were advised to limit screen time and increase their child’s opportunities for social interaction by enrollment in a daycare facility, nursery, or playgroup.

### 2.4. Monitoring for Safety

Before therapy initiation, a complete blood count, kidney function test, and lipid panel were performed, and the levels of electrolytes, liver enzymes, hemoglobin A1c, and prolactin were measured to generate a baseline against which any metabolic side effects of the antipsychotic medications could be monitored [46,47,48,49,50]. Tests were repeated after 3 to 6 months, with subsequent yearly checkups. A fully trained pediatric cardiologist performed an echocardiogram and electrocardiogram on all patients when the maximum dose was reached or earlier if indicated [51,52,53]. At every office visit, serial measurements of weight, body mass index (BMI), height, and vital signs were obtained to further monitor for any side effects. Further, the Abnormal Involuntary Movement Scale (AIMS) was completed at every visit for each patient [54].

### 2.5. Statistical Analysis

Differences between the ordinal data in this study (pre- and post-treatment CGI-S and CARS2-ST scores) were evaluated with the Wilcoxon signed-rank test. A two-tailed *p*-value < 0.05 was considered statistically significant. Data were analyzed using the VassarStats online suite of statistical tools (www.vassarstats.net (accessed on 15 January 2021)).

## 3. Results

Ten cases of ASD were included in this case study (8 males and 2 females) aged 2 to 3½ years (mean age, 2 years 10 months) at presentation. Eight families (80% of patients) opted to initiate treatment with risperidone, whereas two families (20% of patients) opted for aripiprazole, although one patient had to be switched later to aripiprazole because of a lack of response to risperidone. Besides the expected improvement in the patients’ comorbid challenging behaviors, 6/10 patients (cases 1, 2, 6, 8, 9, and 10) achieved a CGI-I score of 1 and minimal-to-no symptoms as per CARS 2-ST, along with complete resolution of their ASD signs and symptoms as per clinical evaluation. Three cases are currently off all medications (cases 6, 8, and 10), while two patients (cases 1 and 2) are being slowly weaned off medication and another patient (case 9) has been kept on ADHD medication to control hyperactivity. The remaining 4/10 patients have attained a CGI-I score of 2 thus far. Patients were weaned off risperidone or aripiprazole very slowly once they had achieved and maintained a CGI-I score of 1 for at least 6 months to prevent adverse effects arising from long-term use. All patients have been monitored for any regression, but none has been observed to date.

The differences in the mean CGI-S and CARS2-ST scores pre- and post-treatment were statistically significant (Z = 2.78, *p* < 0.01 for both) using the Wilcoxon signed-rank test, with pre- and post-treatment median values of 5.5 and 1 for CGI-S, respectively, and 36 and 17 for CARS2-ST, respectively. All medication regimens are listed in Table 1. Each patient’s initial diagnosis according to the DSM-5 criteria and CARS2-ST, initial presenting and current signs and symptoms, baseline and current CARS2-ST and CGI-S scores, and current CGI-I score are provided in Table 2 and Table S1.

Among the seven patients treated with risperidone and with or without methylphenidate or atomoxetine, five patients (71%) achieved a CGI-I score of 1 with complete or almost complete resolution of their ASD signs and symptoms (cases 1, 6, 8, 9, and 10). The other two patients achieved a CGI-I score of 2 (cases 3 and 7). Four patients who achieved a CGI-I score of 1 with complete resolution of their ASD signs and symptoms are currently off risperidone after they had been on risperidone for a minimum of 20 months (cases 6, 8, 9, and 10). One patient (case 5) who did not respond to initial treatment with risperidone was switched to aripiprazole, with clear improvement.

Among the three patients treated with aripiprazole and with or without atomoxetine or methylphenidate, one patient (case 2) attained a CGI-I score of 1 (33%) and is currently weaning off medication, and the other two patients achieved a CGI-I score of 2 (cases 4 and 5).

The laboratory findings were all within normal limits for the patients’ age range. Two patients (20%; cases 8 and 9) developed asymptomatic elevated prolactin levels that were treated conservatively following consultation with a pediatric endocrinologist. Cardiac evaluations did not uncover any adverse cardiac effects. Serial AIMS tests were also performed for every patient at each visit to monitor for any medication-induced abnormal movements but no patient developed any abnormality.

The main side effects noted for risperidone and aripiprazole were weight gain (3/10) and asymptomatic elevated prolactin (2/10). Methylphenidate use caused irritability in 2/10 patients and atomoxetine was associated with aggression in 1/10 patients. Other side effects included mild sedation and drooling that were mainly observed at the start of treatment and later resolved with the continuation of treatment. Weight gain (increase in BMI to > 25) was excessive in two patients (cases 2 and 3). Weight was controlled in both patients by consultations with a nutritionist, diet, and the addition of atomoxetine or methylphenidate where required to improve attention and hyperactivity, as these medications are known to also suppress appetite. One patient (case 2) continued to gain weight with mild inattention on atomoxetine 40 mg and aripiprazole 4 mg at night; therefore, methylphenidate 2.5 mg was included in the morning to help control her weight and improve her attention further.

## 4. Discussion

Our study found that in addition to improving comorbid behaviors, an improvement was observed in the ASD core signs and symptoms for all 10 patients following treatment with risperidone or aripiprazole, with complete resolution of ASD signs and symptoms observed in 60% of patients. This is the first study to ever report the complete resolution of ASD signs and symptoms in patients initiating treatment at less than 4 years of age. It is, however, the second study to show that ASD can be successfully treated—our recent study described the use of a similar treatment approach for 18 children aged between 4 and 10 years old (mean age, 5.7 years) [45]. While many studies have reported some improvement in ASD core signs and symptoms in very young children, none have reported the complete resolution of ASD signs and symptoms [29,30,31,32,33,34,35,37].

There is increasing evidence that early diagnosis (<3 years of age) and prompt treatment are crucial to improve the prognosis of children with ASD [3]. This may be attributable to factors unique to early childhood such as the particularly high levels of synaptogenesis and synaptic pruning that occur within the first 3 years of life. Indeed, aberrant neuronal connectivity and plasticity appear to be the underlying cause of ASD [3,55,56,57,58]. Therefore, interventions should ideally be applied early in life before aberrant neural networks are formed, such that the core signs and symptoms hampering early development are targeted and long-term outcomes are improved.

However, there are currently no medications approved by regulatory bodies for the treatment of the core signs and symptoms of ASD, with risperidone and aripiprazole being the only medications approved by the FDA for treatment of the comorbid symptoms of irritability and aggression, but only in children with ASD from the ages of 5 and 6 years, respectively [11]. In this case study, risperidone or aripiprazole was started at a younger age (2 to 4 years) than that recommended by the FDA. The off-label use of risperidone, aripiprazole, and other psychotropic medications has become an increasingly common practice, particularly in preschool children with ASD [59,60,61,62,63,64,65,66,67]. Unfortunately, clinically randomized controlled trial data for psychopharmacological interventions for ASD in very young children remain particularly rare, lagging behind experience from clinical practice [61,62,66,68]. As a result, parents and clinicians have to carefully weigh the potential benefits and risks of medications against the risks of not intervening when patients are unresponsive to non-pharmacological therapies.

The early and chronic administration of risperidone or aripiprazole with or without methylphenidate or atomoxetine resulted in marked improvement of the CGI-I scores of our patients to between 2 and 1, with complete resolution of the signs of ASD in 60% of patients. Among the seven patients treated with risperidone, five (71%) achieved a CGI-I score of 1, while only one of the three patients (33%) treated with aripiprazole attained a CGI-I score of 1. Although the number of cases in this study is too small to reach any definitive conclusion, these findings suggest that risperidone may offer superior efficacy in treating ASD core signs and symptoms.

There have been numerous studies published on the use of risperidone in the treatment of challenging behaviors in very young children with autism and other pervasive developmental disorders (PDDs), and several of these have also shown benefits in ASD core signs and symptoms in addition to improvements in comorbid challenging behaviors.

For example, Schwam et al. (1998) successfully treated food refusal in a 3-year-old autistic boy [28], and Posey et al. (1999) observed increased eye contact and social relatedness in addition to a marked reduction in persistent and severe aggression and irritability in two autistic boys aged 23 and 29 months who had not responded to non-pharmacological interventions [29]. One child presented with persistent tachycardia and QTc interval prolongation that disappeared following dose reduction. Improved eye contact and social relatedness were also commonly observed among our patients following treatment. Masi et al. (2001) performed a 16-week open-label trial of risperidone in 10 children aged 3.75–6.5 years with PDDs [30]. Only one child was under the age of 4 years, and her parents requested her withdrawal from the study after 20 days of treatment as she had two acute episodes of high fever, inertia, fatigue, and crying; although her parents noted dramatic improvements in her self-injurious behaviors and social relatedness. These improvements regressed to baseline after the discontinuation of risperidone. In the same year, Masi et al. reported a larger open-label study of 24 children with PDDs, aged 3.6–6.6 years; five of these patients were between the ages of 3 years 6 months and 3 years 11 months [31]. While 8/22 patients who completed the trial were rated as much improved to very much improved based on the CGI-1 scale, only one of these responders was under the age of 4 years; 2/5 of the other very young patients were rated as minimally improved, one as unchanged, and one was withdrawn because of tachycardia.

Similar to our report, other studies of very young children have noted improvements in ASD symptoms besides eye contact and social relatedness. In 2002, Boon-Yasidhi et al. reported the treatment of five children aged 2.1–3.7 years with 0.25–0.5 mg risperidone per day [32]. The researchers noted clinically relevant improvements in comorbid behaviors, as well as in speech, eye contact, responsiveness, repetitive behavior, social behavior, and cooperation with supportive therapies. None of the previously reported side effects for risperidone were observed and all children had normal liver and cardiac function.

In 2002, Diler et al. carried out a 6-month open-label study of risperidone among 20 children with ASD aged 3–7.5 years [33]. Three of the children were less than 4 years of age but two were withdrawn from the study because of non-compliance to the study protocol or the presence of severe irritability, anger, and aggression that disappeared a few days after treatment cessation. The remaining patient showed no adverse effects and had an improvement in her CGI-S score from 5 to 4. A 3-year naturalistic study by Masi et al. (2003) of 53 children with autism and other PDDs aged 3.6–6.6 years considered 22/47 (47%) patients who continued with risperidone for at least 1 month to be responders [34]. The main reason for discontinuation of treatment was an elevated prolactin level, although this was asymptomatic in all cases as was observed for two of our patients.

Further, Nagaraj et al. (2006) performed a randomized, placebo-controlled, and double-blinded study of 40 children with ASD aged 2–9 years who were treated with risperidone or a placebo [35]. Among those receiving risperidone, 12/19 and 17/19 had significant improvements in their total CARS score and Children’s Global Assessment Scale score, respectively. It is unclear though how many of these patients were aged less than 4 years. In 2014, Boon-Yasidhi et al. specifically investigated the adverse effects of risperidone in a cross-sectional study of 45 children with ASD, aged 2–15 years [36]. Adverse effects were observed in 39/45 children, but these were mostly mild and tolerable. Lastly, Fayyazi et al. (2014) investigated the use of risperidone and buspirone in the treatment of severe behavioral problems associated with phenylketonuria in 42 children aged 2–6 years [37]. Six of these children were diagnosed with ASD. Risperidone was found to effectively control hyperactivity, as well as disruptive and stereotypic behaviors.

Among our patients, the adverse effects were either mild and transient (e.g., sedation and drooling) or responsive to adjunct medication (i.e., methylphenidate or atomoxetine for excessive weight gain and inattention). Where possible, however, the use of methylphenidate or atomoxetine was avoided as these medications may add further to the potential burden of adverse effects and their use is off-label in very young children. The main side effects noted for risperidone and aripiprazole were weight gain (30%) and asymptomatic elevated prolactin (20%), which have been noted in previous studies [11,19,69].

Notably, three patients (cases 1, 6, and 7) who initiated treatment at a particularly early age with risperidone or aripiprazole (around 2 years old) showed especially robust improvement and started to communicate and connect with their environment, caregivers, and others within a very short time. These patients were described by their family as if they were initially “living in a bubble and suddenly got out of their bubble” once the medication was initiated. They tolerated their medication well without unusual side effects at this early age.

It is possible that the improvements we observed in our patients’ core signs and symptoms of ASD, which most previous studies with older children failed to demonstrate [70], might have been influenced by treatment earlier in life, the chronic administration of such medications, and the use of a highly individualized approach of adding ADHD medication to augment attention when needed, adjusting the dose according to indications of efficacy and safety, and switching medication for non-responders. Further, the use of standard supportive therapies by our patients may have assisted them in attaining the observed improvements: however, these therapies have never been reported to completely resolve ASD core signs and symptoms.

This is the first study to ever report the complete resolution of ASD signs and symptoms in patients initiating treatment at less than 4 years of age. It is, however, the second study to show that ASD can be successfully treated—our recent study described the use of a similar treatment approach for 18 children aged between 4 and 10 years old (mean age, 5.7 years) [45]. While many studies have reported some improvement in ASD core signs and symptoms in very young children, none have reported the complete resolution of ASD signs and symptoms.

This retrospective case study had the following limitations: it was open-label in nature without any controls or randomization; the confounding effects of non-pharmacological interventions were, therefore, not controlled; and the patients did not follow a unified comprehensive treatment model or focused intervention model [13]. However, despite these shortcomings and the small number of cases included, we believe our unprecedented findings regarding the complete resolution of ASD core signs and symptoms in very young children, as well as the treatment protocol used, warrant reporting. While firm conclusions cannot be drawn because of these study limitations, it is hoped our findings will stimulate further research in this area. Importantly, the long-term effects of antipsychotic use, particularly on the developing brain, are currently unknown in such a young population, and this requires further investigation.

## 5. Conclusions

This report presents a retrospective case study of 10 children who showed marked improvement in their core ASD signs and symptoms to grade 1 or 2 on the CGI-I scale with early and chronic administration of risperidone or aripiprazole, and who initiated treatment between the age of 2 to 4 years. Although limited by a small number of cases, our findings suggest that antipsychotic medication started early in life can potentially eliminate the core signs and symptoms of ASD. This is consistent with increasing evidence that early intervention is critical for improved long-term outcomes for patients with ASD. It is hoped that sufficiently powered, double-blind, and placebo-controlled trials will be conducted in the future to verify these findings in this pediatric population.

## Figures and Tables

**Table 1:** Clinical characteristics of patients.

Case No.	Sex	Age (Years); Date Medication Started (mm/yy)	Medication Starting Dose (mg)	Maximum Dose (mg); ^a^ Time to Reach this Dose	Current Dose (mg); ^a^ Total Duration	Laboratory Findings ^b^	Cardiac Evaluation	Medication and Side Effects
1	M	2; 06/19	Risperidone 0.25 mg at night.	Risperidone 2 mg in the morning and 0.5 mg at night; 8 months.	Risperidone 2 mg in the morning and 0.5 mg at night; 15 months.	Normal	Normal	Currently weaning off risperidone.
2	F	3; 03/19	Aripiprazole 1 mg at night. Atomoxetine 10 mg added at night after 1 month. Methylphenidate 2.5 mg added in the morning after 3 months.	Aripiprazole 4 mg at night; 1 month. Atomoxetine 40 mg at night; 3 months. Methylphenidate 2.5 mg in the morning; 3 days.	Aripiprazole 4 mg at night; 21 months. Atomoxetine 40 mg at night; 18 months. Methylphenidate 2.5 mg in the morning; 16 months.	Normal	Normal	Weight gain on aripiprazole; therefore, atomoxetine, and later methylphenidate, were added. Methylphenidate was tried for 3 days at 5 mg but caused irritability so it was reduced back to 2.5 mg. Currently weaning off aripiprazole.
3	M	3; 06/19	Risperidone 0.25 mg at night. Methylphenidate 2.5 mg added in the morning after 3 months.	Risperidone 2 mg in the morning and 0.5 mg at night; 9 months. Methylphenidate 10 mg in the morning and at 2 pm; 5 months.	Risperidone 2 mg in the morning and 0.5 mg at night; 15 months. Methylphenidate 10 mg in the morning and at 2 pm; 12 months.	Normal	Normal	Weight gain on risperidone; methylphenidate was added later to control weight and help attention.
4	M	3; 09/19	Aripiprazole 1 mg at night.	Aripiprazole 10 mg at night; 7 months. Atomoxetine 10 mg at night; one week. Methylphenidate 2.5 mg in the morning; 3 days.	Aripiprazole 10 mg at night; 15 months.	Normal	Normal	One-week atomoxetine 10 mg trial made him very aggressive. Three-day methylphenidate 2.5 mg trial made him irritable.
5	M	3; 07/19	Risperidone 0.25 mg at night. Aripiprazole 1 mg added at night after 5 months.	Risperidone 1.5 mg twice a day; 5 months. Aripiprazole 3 mg at night; 2 months.	Aripiprazole 3 mg at night; 12 months.	Normal	Normal	Risperidone was weaned after 5 months as there was no clear improvement. It was replaced by aripiprazole.
6	M	2⅓; 09/17	Risperidone 0.25 mg at night.	Risperidone 1.75 mg in the morning and 0.5 mg at night; 13 months. He was subsequently weaned off risperidone and was on risperidone for a total of 28 months.	None	Normal	Normal	
7	M	2; 12/19	Risperidone 0.25 mg at night.	Risperidone 1 mg in the morning and 0.75 mg at night; 4 months.	Risperidone 1 mg in the morning and 0.75 mg at night; 12 months.	Normal	Normal	
8	M	3½; 09/17	Risperidone 0.25 mg at night.	Risperidone 2 mg in the morning and 0.5 mg at night; 8 months. He was subsequently weaned off risperidone and was on risperidone for a total of 20 months.	None	Prolactin was elevated 6-fold with no clinical signs or symptoms of hyperprolactinemia.	Normal	Mild weight gain.
9	F	3⅓; 12/16	Risperidone 0.25 mg at night. Atomoxetine 10 mg added at night after 9 months.	Risperidone 1.5 mg in the morning and 0.75 mg at night; 12 months. She was subsequently weaned off risperidone and was on risperidone for a total of 27 months. Atomoxetine 40 mg; 2 months.	Atomoxetine 40 mg at night only; 48 months.	Prolactin was elevated 2.5-fold with no clinical signs or symptoms of hyperprolactinemia.	Normal	Once she attained normal development and complete resolution of her ASD symptoms she was weaned off risperidone. Kept on atomoxetine to help control hyperactivity only.
10	M	3; 07/18	Risperidone 0.25 mg at night. Atomoxetine 10 mg added at night after 10 months. Methylphenidate 2.5 mg added in the morning after 11 months.	Risperidone 2 mg in the morning and 0.5 mg at night; 24 months. Atomoxetine 10 mg at night stopped after one week due to irritability. Methylphenidate 2.5 mg in the morning stopped after one week due to irritability.	None	Normal	Normal	

ASD, autism spectrum disorder; F, female; M, male. ^a^ All medications were given orally. Risperidone and aripiprazole were provided as a syrup. Short-acting methylphenidate was crushed and given mixed with 5 mL of water. Atomoxetine was given as an oral solution. ^b^ Complete blood count, electrolytes, liver enzymes, kidney function test, lipid panel, hemoglobin A1c, and prolactin.

**Table 2:** Diagnosis, scores at baseline and after treatment, and current status of all 10 patients.

Case No.	Diagnosis: DSM-5 Criteria	CGI-S/CARS2-ST Scores before Treatment	CGI-S/CARS2-ST Scores after Treatment	CGI-I Score after Treatment	Current Status
1	ASD level 2	5/36	1/17	1	Complete resolution of ASD symptoms, currently weaning off medications
2	ASD level 3	6/45.5	1/16	1	Complete resolution of ASD symptoms, currently weaning off medications
3	ASD level 2	6/45	2/23	2	Much improved
4	ASD level 2	5/39.5	2/21	2	Much improved
5	ASD level 2	5/36	2/18.5	2	Much improved
6	ASD level 3	6/42	1/16	1	Complete resolution of ASD symptoms, off all medications
7	ASD level 3	6/41	2/21	2	Much improved
8	ASD level 2	5/36	1/15	1	Complete resolution of ASD symptoms, off all medications
9	ASD level 2	5/36	1/17	1	Complete resolution of ASD symptoms, only on atomoxetine to control hyperactivity
10	ASD level 2	6/36.5	1/15	1	Complete resolution of ASD symptoms, off all medications

ASD, autism spectrum disorder; CGI-I, Clinical Global Impression–Improvement; CGI-S, Clinical Global Impression–Severity; CARS2-ST, Clinical Autism Rating Scale 2–Standard Test.

## Data Availability

Not applicable.

## Supplementary Materials

The following are available online at https://www.mdpi.com/article/10.3390/children8050318/s1. Table S1: Details of each patient’s symptoms at presentation, diagnosis, and current status.
