# Supplementary material for: Risperidone or Aripiprazole Can Resolve Autism Core Signs and Symptoms in Young Children: Case Study
Source: Children (Basel). 2021 Apr 22;8(5):318. doi: 10.3390/children8050318 (PMC8143447; doi:10.3390/children8050318)
Supplement: Supplementary file 1 [file children-08-00318-s001.zip › children-1171467-supplementary.pdf]

Supplementary Table S1 Details of each patient's symptoms at presentation, diagnosis, and current status

Case	Main symptoms at presentation	Diagnosis as per DSM-5 criteria	CGI-S/CARS2-ST scores before treatment	Current behavior after treatment	CGI-S/CGI-I/CARS2-ST scores after treatment
1	- sudden developmental regression at the age of 18 months<br>- lost all social skills<br>- lost eye contact<br>- no response to his name<br>- no joint attention<br>- hand flapping<br>- repetitive touching of ears<br>- walked on his toes<br>- lost interest in playing with other children<br>- no expressive language<br>- poor receptive language	ASD level 2, requiring substantial support, plus language impairment	5/36	- follows two-step commands<br>- started to count<br>- responds to his name consistently<br>- can combine words to form simple sentences (e.g., "key cars go").<br>- jokes with mother<br>- imitates animal sounds<br>- no longer flaps hands<br>- no longer walks on toes or repeatedly touches his ears<br>- started to play with other children and give high-fives<br>- family thinks he is comparable in development to his peers	1/1/17
2	- poor eye contact<br>- no response to her name<br>- no pointing<br>- no joint attention<br>- did not play with other children	ASD level 3, requiring very substantial support, plus language impairment	6/45.5	- can now talk in three- to four-word sentences<br>- makes good eye contact<br>- started to point	1/1/16

	- delayed expressive and receptive language<br>- aggressive, impulsive, and had frequent temper tantrums<br>- was very difficult to manage<br>- very selective in her food and habitually threw things			- intact joint attention<br>- shows no aggression or stereotypical behavior<br>- plays with other children<br>- started to learn the alphabet and numbers<br>- started to eat a greater variety of food<br>- currently weaning off medications	
3	- poor response to his name<br>- poor joint attention<br>- delayed expressive and receptive language<br>- no pointing<br>- did not play with other children<br>- liked to jump on his bed<br>- no emotional reactions<br>- liked to hold orange items<br>- liked to wear only one specific shirt<br>- very hyperactive and inattentive	ASD level 2, requiring substantial support, plus language impairment	6/45	- good response to his name<br>- good joint attention<br>- much improved expressive and receptive language<br>- started to point well<br>- plays well with other children<br>- no longer jumps on his bed<br>- interacts with his family and has started to show emotions<br>- no longer likes to hold orange items<br>- can wear different shirts now<br>- still a little hyperactive and inattentive	2/2/23
4	- poor eye contact<br>- poor expressive and receptive language<br>- abnormal, stereotypical hand and finger movements<br>- self-talked a lot	ASD level 2, requiring substantial support, plus language impairment	5/39.5	- shows good eye contact<br>- responds to his name consistently<br>- has good joint attention<br>- started to play with family members	2/2/21

	- no joint attention<br>- very limited scope of interest<br>- did not play with other children			- speaks in two- to three-word sentences to communicate his needs<br>- started to learn the alphabet<br>- complete resolution of stereotypical behavior such as finger flicking<br>- no longer displays self-talking	
5	- poor response to his name<br>- poor eye contact<br>- poor communication skills<br>- no pointing<br>- self-talked a lot<br>- poor joint attention<br>- very delayed expressive and receptive language<br>- poor social skills<br>- abnormal, stereotypical behavior with hand flapping<br>- hyperactive with a limited attention span	ASD level 2, requiring substantial support, plus language impairment	5/36	- started to focus and sit down for 45 minutes at a time<br>- started to recognize and interact with family members<br>- started to speak in two- to three-word sentences<br>- follows two-step commands<br>- hand flapping is much less, only occurs now when he is very excited<br>- no more self-talking<br>- responds to his name well<br>- developed joint attention<br>- good eye contact	2/2/18.5
6	- poor response to his name<br>- poor eye contact<br>- no pointing<br>- did not play with other children	ASD level 3, requiring very substantial support, plus language impairment	6/42	- achieved normal development with complete resolution of ASD symptoms<br>- now attends a regular school where he performs above average<br>- off all medications	1/1/16

	- hyperactive and poor attention<br>- no joint attention<br>- poor expressive and receptive language<br>- stereotypical behavior of walking on his toes and looking from eye corners				
7	- poor eye contact<br>- no joint attention<br>- no pointing<br>- did not follow commands<br>- poor expressive and receptive language<br>- did not play with other children<br>- no meaningful communication with his parents<br>- very sound sensitive<br>- very hyperactive and inattentive<br>- poor sleep at night	ASD level 3, requiring very substantial support, plus language impairment with comorbid ADHD	6/41	- much less hyperactive<br>- started to count to 10<br>- started to communicate with parents and follow one-step commands<br>- started to say his name when asked<br>- started to approach other family members<br>- started to have better receptive language<br>- makes good eye contact<br>- good joint attention<br>- no longer sound sensitive<br>- started to sleep well at night	2/2/21
8	- poor response to his name<br>- no pointing<br>- no expressive language with poor receptive language<br>- no joint attention	ASD level 2, requiring substantial support, plus language impairment	5/36	- attending regular school and performs above average academically<br>- complete resolution of his ASD symptoms<br>- off all medications	1/1/15

	- poor socialization with other children<br>- walked on his toes<br>- ate only two kinds of food<br>- flapped his hands when excited<br>- hypersensitive to sounds<br>- hyperactive with a poor attention span				
9	- poor eye contact<br>- no response to her name<br>- no joint attention<br>- no pointing<br>- poor social interaction and did not play with other children<br>- no joy in sharing<br>- very selective with her food<br>- liked to line things up<br>- high tolerance to pain<br>- had poor expressive language, could only say “mama” and “dada” non-specifically, and had poor receptive language with difficulty in following commands<br>- poor attention and hyperactive	ASD level 2, requiring substantial support, plus language impairment	5/36	- attends regular school and among the top 5 in the class<br>- normal expressive and receptive language<br>- normal behavior and attention span<br>- complete resolution of ASD symptoms<br>- only on atomoxetine to control hyperactivity	1/1/17
10	- poor eye contact	ASD level 2, requiring	6/36.5	- now in a regular school	1/1/15

	- poor joint attention<br>- did not play with other children<br>- poor expressive and receptive language<br>- rarely pointed<br>- hyperactive and inattentive<br>- no joy in sharing<br>- frequently stared into the distance and at the ceiling<br>- no imagination<br>- anxiety and temper tantrums	substantial support, plus language delay and comorbid anxiety		- average performance in class<br>- normal expressive and receptive language<br>- complete resolution of ASD symptoms<br>- still below average in writing skills but improving with training	

ADHD, attention deficit hyperactivity disorder; ASD, autism spectrum disorder; CGI-I, Clinical Global Impression–Improvement; CGI-S, Clinical Global Impression–Severity; CARS2-ST, Clinical Autism Rating Scale 2–Standard Test
